# Supplementary material for: Phosphorylation-Mediated Activation of β-Catenin-TCF4-CEGRs/ALCDs Pathway Is an Essential Event in Development of Aggressive Hepatoblastoma
Source: Cancers (Basel). 2022 Dec 9;14(24):6062. doi: 10.3390/cancers14246062 (PMC9775972; doi:10.3390/cancers14246062)
Supplement: Supplementary file 1 [file cancers-14-06062-s001.zip › cancers-2065537-Supplementary.pdf]

cancers

MDPI

Supplementary Materials: Phosphorylation-Mediated Activation of β-Catenin-TCF4-CEGRs/ALCDs Pathway Is an Essential Event in Development of Aggressive Hepatoblastoma

Ruhi Gulati, Margaret A. Hanlon, Maggie Lutz, Tyler Quitmeyer, James Geller, Gregory Tiao, Lubov Timchenko and Nikolai Timchenko

Supplemental Methods

Mutational analysis of exon 3 of β-catenin gene. The presence of the deletion/mutations in exon 3 of β-catenin gene, which is known to be often mutated in patients with HBL, were examined in specimens from HBL patients, used in these studies (Supplemental figure 1). PCR for human CTNNB1 was performed using Phusion High-Fidelity PCR Mastermix (ThermoFisher Scientific F53L) following manufacturer's instructions.

Western Blot Analysis of GPC3. For investigations of GPC3, two modified protocols of protein isolation were used to protect GPC3 from proteolytic cleavage/degradation which occurs during isolation of the proteins. As shown on the Supplemental Figure S2, GPC3 undergoes cleavage/degradation in protein extracts from HBL patients. Therefore, a shorter time (10 min) incubation with buffer B and a shorter time of centrifugation (5 min) were applied. In the second protocol, we performed homogenization of tissues/cells in SDS-loading buffer containing 2% SDS and 5mM B-mercaptoethanol and the mixture was boiled for 40 min. After centrifugation for 10 min, supernatant was used for Western Blot analysis. Both protocols provided non-degraded 70kD GPC3 protein (Supplemental Figure S2). In some HBL cases, an additional isoform of GPC3 with MW 90kD was observed.

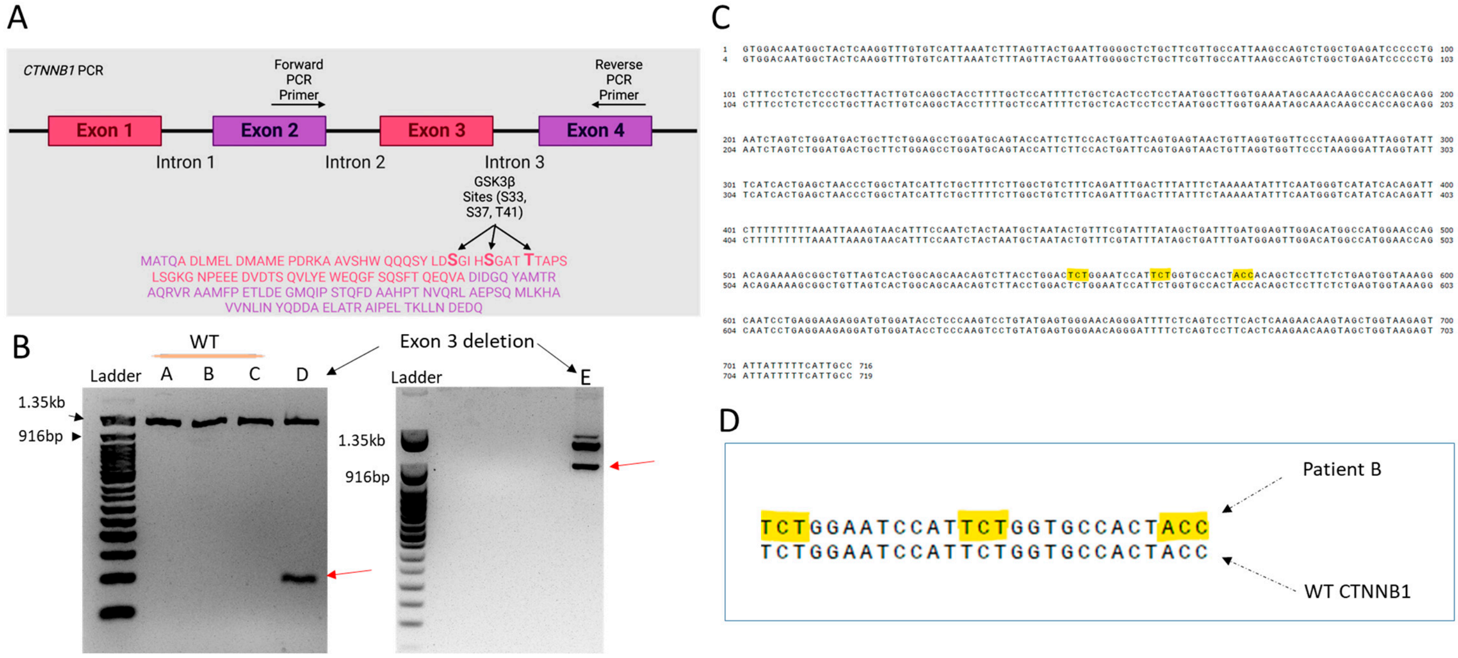

Figure S1. Analysis of deletions/mutations in exon 3 of CTNNB1 gene. **A)** A strategy for detecting CTNB1 mutations. PCR primers are predicted to produce 1148bp DNA fragment with exon 3 in the middle. Below shows amino acid sequence of WT exons 2, 3, and 4 **B)** Examples of PCR products with and without deletion of exon 3.**C)** Sequence alignment of HBL Patient B (top sequence) to WT CTNNB1 (bottom sequence). Highlighted codons encode S33, S37 and T41. Primer sequences and PCR conditions were obtained from Nhieuet al. 1999 Am J Pathology. **D)**Sequences of regions of CTNNB1 in patient B and in WT CTNNB1 which code for S33-S37-T41.

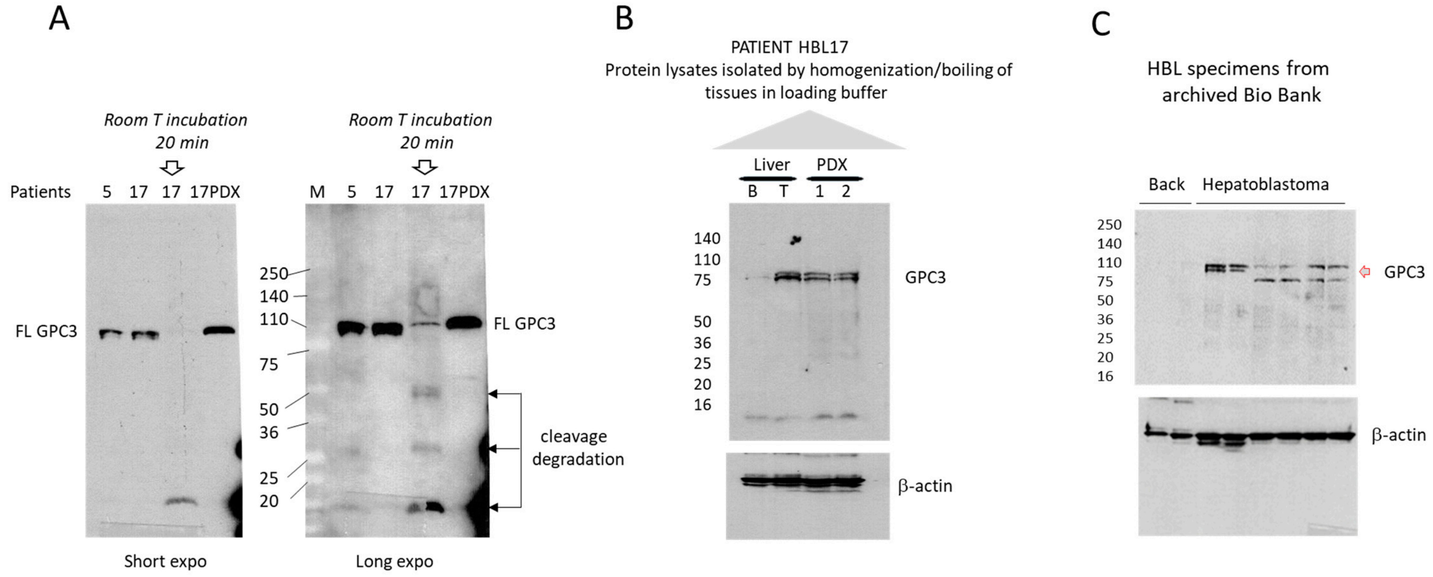

Figure S2. Improvements of protocols for isolation of non-degraded GPC3. **A)** Protein extracts contain an enzyme (s) which cleaves/degrades GPC3. WCEs were isolated by a fast procedure (described in the Methods) from HBL5, HBL17 and PDX of patient 17. WCE from patient 17 was left on room temperature for 20 min before loading. Short and long exposures are shown. **B)** Homogenization/boiling of tissues in SDS sample buffer prevents degradation of GPC3. Tissues from background and tumor sections of HBL patient 17 and tumors from PDXs were homogenized in an SDS-sample buffer, boiled for 40min and loaded on the gel. **C)** Western blot analysis of GPC3 with archived HBL samples using homogenization/boiling protocol. GPC3 is detected as 70kD and 90kDisoforms.

CUGBP1 Sequence 2: chr11:47534882-47535299
CGCAGTGGCTCACGCCTGTAATCCTAGCACTTTGGGAGGCCGAGGTGGGC
GGATCACGAGGTCAGGAGATCGAGACCATCCTGGCTAACATGGTGAAACC

P53 Sequence 2: chr17:7598141-7598558
GCTCACGCCTGTAATCCCAGCACTTTGAGAGGCCAAGGCGGGCGGATCAC
GAGGTCAGGAGATCGAGACCATCCTGGCTAACATGGTGAAACCCCGACTG

C/EBPα Sequence: chr19:33793460-33793877
TGGTTCTGGCTTTGAAAGAGAATCCGCGCCCCAGCAGCTCAAGACCAAG
ACTCGCCCTCCGCCCCCCACCCCTACCCCGTGCAGCCTCGGGATACTCCT

RB Sequence: chr13:48887761-48888172 76% CONSERVED
GTGGCTCACGCCTGTAATCCCAGCACTTTGGGAGGCCGAGGTGGGCGATC
ACGAGGTCAGGAGATCGAGACCATCCTGGCTAACACAGTGAAACCCCGTC

RUNDC1: chr17:41138385-41138802
AGCTCACGCCTGTAATCCCAGCACTTTGGGAGGCCGAGGCGGGTGGATCA
CGAGGTCAGGAGATTGACCATCCTGGCTAACACAGTAAAACCCCGTCTCT

PMPCA: chr9:139315732-139316149
GCTCCCGCCTGTAATCCCAGCTCTTTGGGAGGCTGAGGCAGGCGGATCAC
GAGGTCAGGAGATTGAGACCATCCTGGCTAACAGAGTGAAACCCCTTCTC

PGAP1: chr2:197759256-197759673
GTGGCTCATGCCTGTAATTCCAGCACTTTGGGAGGCCAAGACGGGTGGAT
CACAAGGTCAGGAGATCGAGACCATCCTGGCTAACACGTAGAAACCCTGT

HACE1: chr6:105221835-105222252
TGGCTCACGCCTGTAATCCCAGCACTTTGGGAGGCCGAGGCGGGCGGATC
ACGAGGTCAGGAGATCGAGACCATCCCGGCTAAAACGGTGAAACCCCGTC

FAM46D: chrX:79597081-79597498
GTGGCTCACGCCTGTAGTCCCAGCACTTTGGGAGGCCGAGGCGGGCGGAT
CACGAGGTCAGGAGATCGAGACCATCCTGGCTAACACGGTGAAACCCCGT

KRT86: chr12:52675887-52676304
CTCACGCCTGTAATCCCAGCACTTTGGGAGGCTGAGGCGGGCGGATCACG
AGGTCAGAAGATCGAGACCATCCTGGCTAACACAGAGAAACATCGTCTCT

WNT98: chr17:44960215-44960632
CTCACGCCTGTAATCCCAGCACTTTGGGAGGCTGAGGTGGGCGGATCACG
AGGTCAGGAGATCAAGACCATCCTGGCTAACGTGGTGAAACCCCGTCTCT

ARSG: chr17:66296989-66297406
CAAGCTTTAAAAAAAAAAAAAAAGCCACTTTGGGAGGCCAAGGTGGGTAGAT
CACGAAGTCAGGAGATCGAGACCATCCTGGCTAACATGGTGAAACCCCGT

ACBD5: chr10:27505805-27506222
GTGGCTCACGCCTGTAATCTCAGCACTTTGGGAGGCAGAGGAGGGCAGAT
CACAAGGTCAGGAGATCGAGACCATCCTGGCTAACACGGTGAAACCCCGT

GSN: chr9:123965135-123965552
TGGCTCACGCCTGTAATCCCAGCACTTTGGGAGGCCACGACGGGCGGATC
ACGAGGTCAGGAGATCGAGACCATCTTGGCTAACACGGTGAAACCCCGTC

ANKH: chr5:14721769-14722186
GGTGGCTCATGCCTGTAATCCAGCACTTTGGGAGGCCAAGGTGGGAGGAT
CACAAGGTCAGGAGATCGAGACCATCCTGGCTAACACGGTGAAACCCCAT

GABRA5: chr15:27130176-27130593
GCTCACGCCTGTAATCCCAGCACTTTGGGAGGCCGAGGCGGGAGGATCAC
GAGATCAGGAGATCGAGACCATCCTGGCTAACTTGGTGAAACCCCGTCTC

Figure S3. Nucleotide sequences of 5′regions of CEGRs/ALCDs in cancer genes contain a strong binding site for a partner of β-catenin TCF4. Red color shows TCF4 binding sites. A core CTTTG sequence is underlined.

YEATS2: chr3:183446790-183447207
GTGGCTCACACCTATAATCCAAGCACTTTGGGAGGCCAAGGCAGGCGGAT
CATGAGGTCAGGAGATTGAGACCATCCTGGCTAACACGGTGAAACCCGGT

LRRC37A3: chr17:62913094-62913511
TGGCTCACGTCTGTAATCCCAGCACTTTGGGAGGCCGAGGCGGGCAGATC
ACGAGGTCAGGATATCGAGACCATCCTCGCTAACATGGTGAAACCCCGTC

RPS29: chr14:50041629-50042046
GCTTACGCCTGTAATCCTAGCACTTTGGGAGGCTGAGGCGGGTGGATCAC
CTGAGGTCAGGAGTTAAAGACCAGCCTGGCCAATGCAGTGAAACCCCATC

TM4SH19: chr3:196052716-196053133
TGGCTCACGCCTGTAATCCCAGCACTTTGGGAGGCCAAGATGGGCGGATC
ACCTGAGGTCAGGAGTTTGAGACCAGCCTGGCCAACATGGTGAAACCCCG

MAST1: chr19:12959171-12959588
GGCCTGTAATCCCAGCACTTTGGGAGGCCGAGGGGGGCGGATCACGAGGT
CAAGAGATCGAGACCATCCTGGCTAACACGGTGACACCCCGTCTCTACTA

PALM2-AKAP2: chr9:112686610-112687027
AGATGGTGCCTGTAATCCCAGCACTTTGGGAGGCCGGGGCGGGTGGATCA
CGAGGTCAGGAGATCGAGACCATCCTGGCTAACGTGGTGAAACCCTGTCT

FAM120A02: chr9:96210770-96211187
CACGCCTGTAATCCCAGCACTTTGGGAGGCTGAGGTGGGCGGATCACAAG
GTCAGGAAATCCAGACCATCCTGGCTAACACGGTGAAACTCCGTCTCTAC

MYO18B: chr22:26177307-26177724
TGGCTCACGCCTGTAATCCCAGCACTTTGGGAGGCTGAGACGGGTGGATC
ATGAGGTCAGGAGATTGACACTATCCTGGCTAATGCGGTGAAACCCCGTC

HDAC1
GCACTTTGGGAGGCCGAGGCGGGCAGATCACGAGGTCAGGAGATCAAG
ACCATCCTGGCTAACACAGTGAAACCCCATCTCTACTAAAAATACAAAAA

FBXL18: chr7:5514209-5514626
TGGCTCACGCCTGTCATCCCAGCACTTTGGGAGGCCGAGGCGGGCGGATC
ACGAGGTCAGGAGATCAAGACCACCCTGGCGAACACGGTGAAACCCCGTC

LINC02242: chr5:66928100-66928517
GTGGCTCACGCCTGTAATCCCAGCACTTTGGGAGGCCGAGGCGGGCGGAT
CACGAGGTCAGGAGATCGAGACCATCCCGGCTAAAACGGTGAAACCCCGT

SIDT1: chr3:113287171-113287588
GGTGGCTCACTCCTGTAATCCTAGCACTTTGGGAGGCCGAGGAGGTGGAT
CACGAGGTCAGGAGATCGAGACCATCCTGGCTAACACAGTGAAACCCCAT

TSPAN14: chr10:82252302-82252719
GTGGCTCACATCTGTAATCCCAGCACTTTGGGAGGCCGAGGCAGGCGGAT
CACGAGGTCAGGAGATCAAGACCATCCTGGCTAACACGGTGAAACTCCGT

C11orf65: chr11:108256139-108256556
GTGGCTCTCGCCTGTAATCCCAGCACTTTGGGAGGCCGAGGTAGTTGGAT
CACAAGGTCAGCAGATCAAGACCATCCCGGCTAACACAGTGAAACCCTGT

GSS: chr20:33541289-33541706
TGGGCGGGGTGGCTCACGCCTGTAATCCCAGCACTTTGGGAGGCCGAGAG
TGGGGGATCACGAGATCGAGACCATCCTGGCTAACACGGTGAAACCCCGT

TRIM16L: chr17:18637497-18637914
GTGGCTCACGCCTGTAATCCCAGCACTTTGGGAGGCCGAGGCGGGTGGAT
CATGAGGTCAGGAGATCGAGACCATCCTGGCTAACAAGGTGAAACCCCGT

TNFRSF19
CCTGTAATCCCAGCACTTTGGGAGGCCGAGGCGGGCGGATGGTCA
GGATCAAGACCATCCTGGCTAACATGGRGAAACCCCGTCCCTACTAC

HMG2A: chr12:65822460-65915527
gcctgtaat***cccagcactttggg***aggccgaggcgggtggatcatgaggtcagga
gatcgagaccatcctggctaacaaggtgaaaccccgtctcta

Figure S4. Nucleotide sequences of 5′regions of CEGRs/ALCDs of additional genes containing a strong binding site for a partner of β-catenin TCF4. Red color shows TCF4 binding sites. A core CTTTG sequence is underlined.

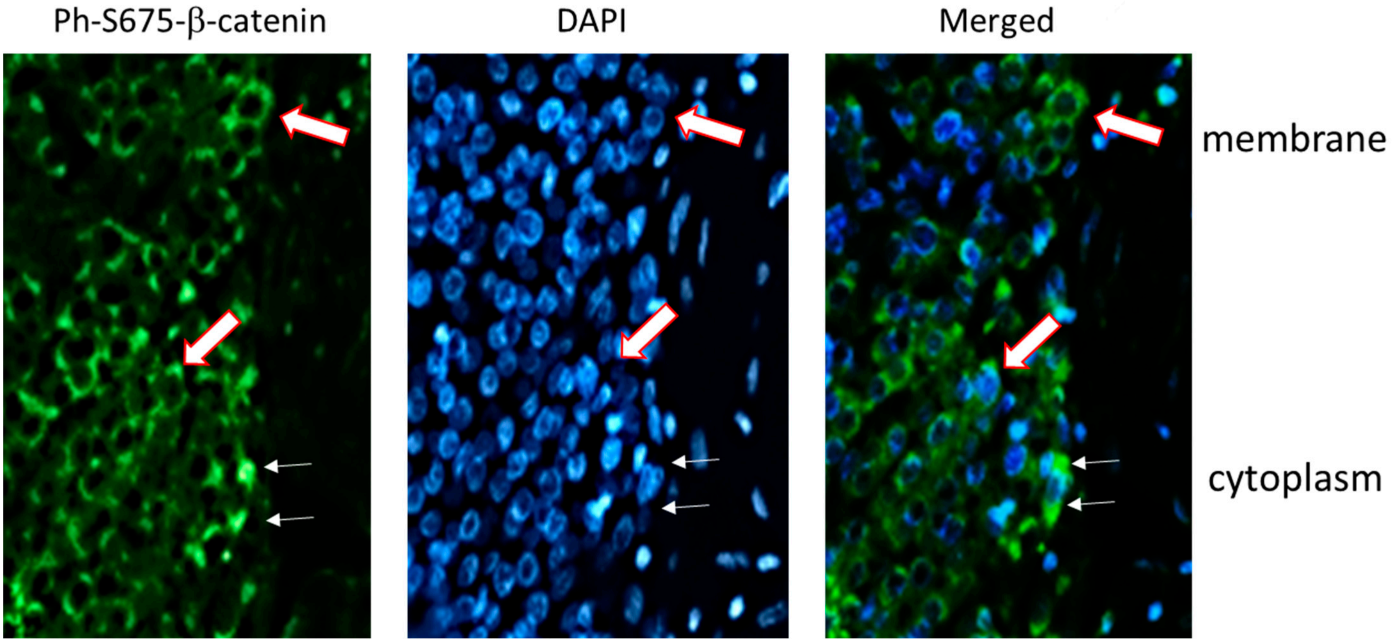

Figure S5. A typical image of staining of HBL tumor #85 with Abs to ph-S675-b-catenin. Open red arrows show staining on cell membranes. White arrows show examples of cytoplasmic staining.

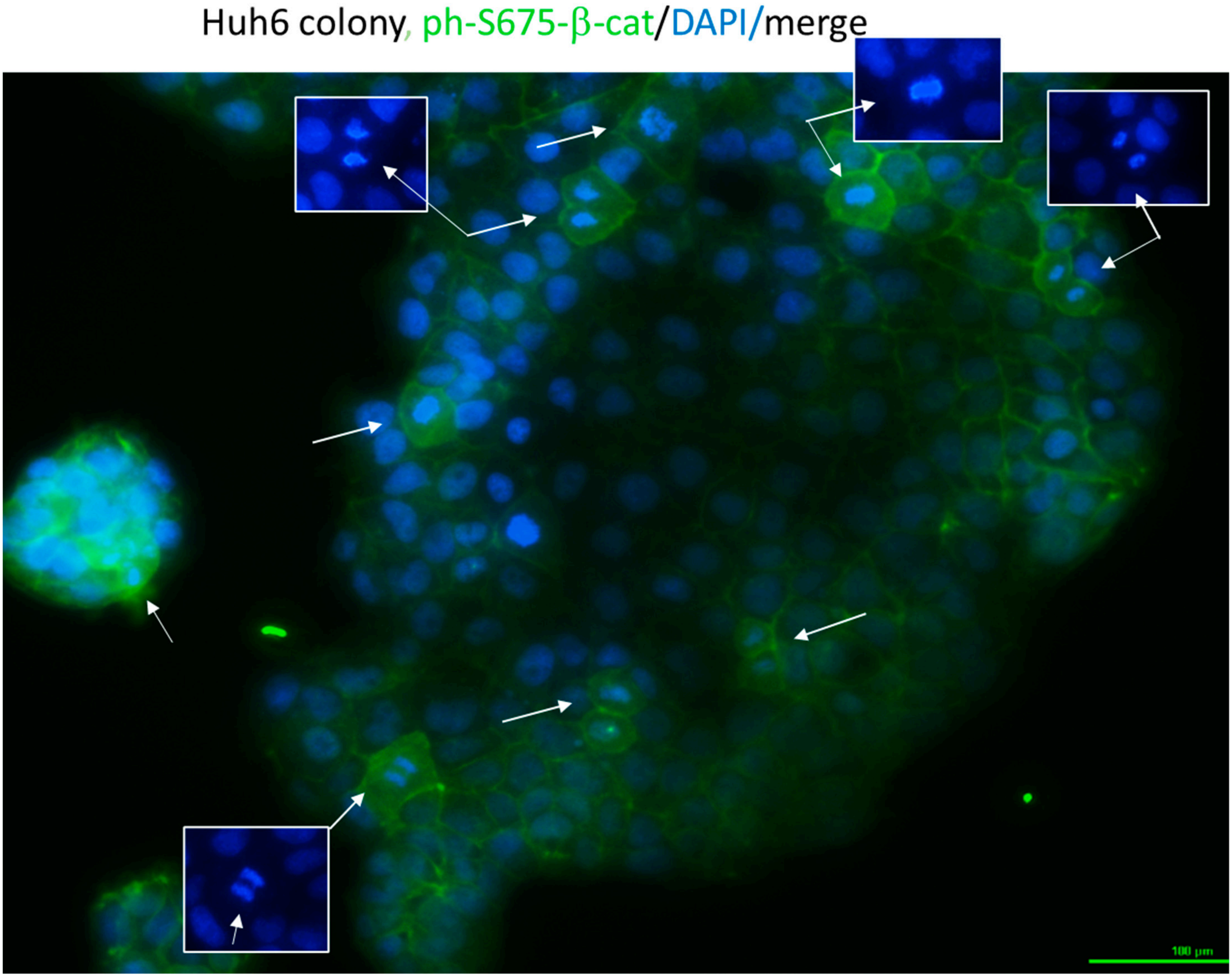

Figure S6. A large field of staining of Huh6 cells with ph-S675-β-catenin. Arrows show mitotic figures in cells with strong ph-S675-β-catenin staining. Large boxes show DAPI staining of cells where mitotic figures are clearly seen.

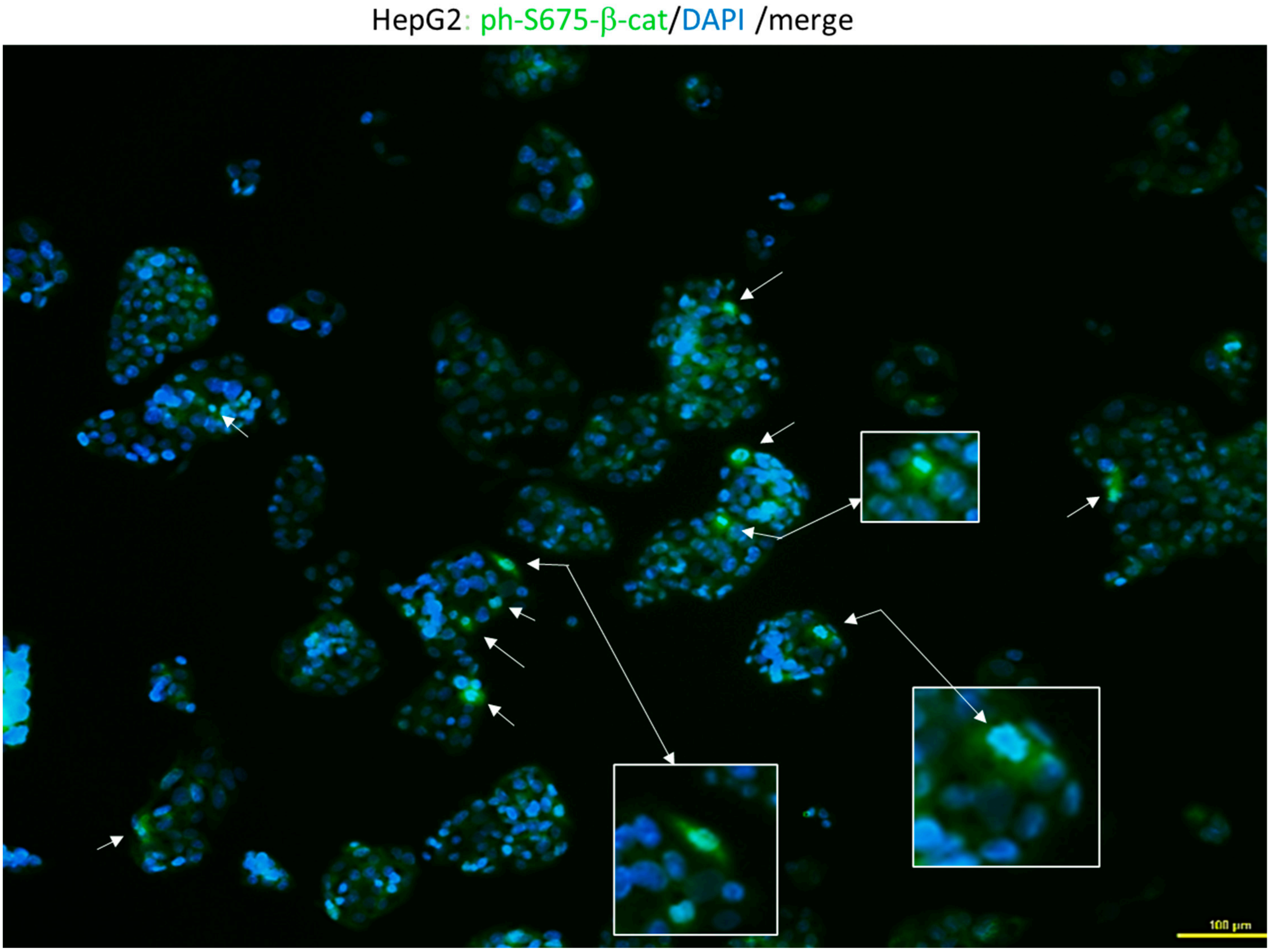

Figure S7. A large field of staining of HepG2 cells with ph-S675-β-catenin. Arrows show mitotic figures in cells with strong ph-S675-b-catenin staining mainly in nuclei. Large boxes show merge staining of cells where nuclear staining and mitotic figures are clearly seen.

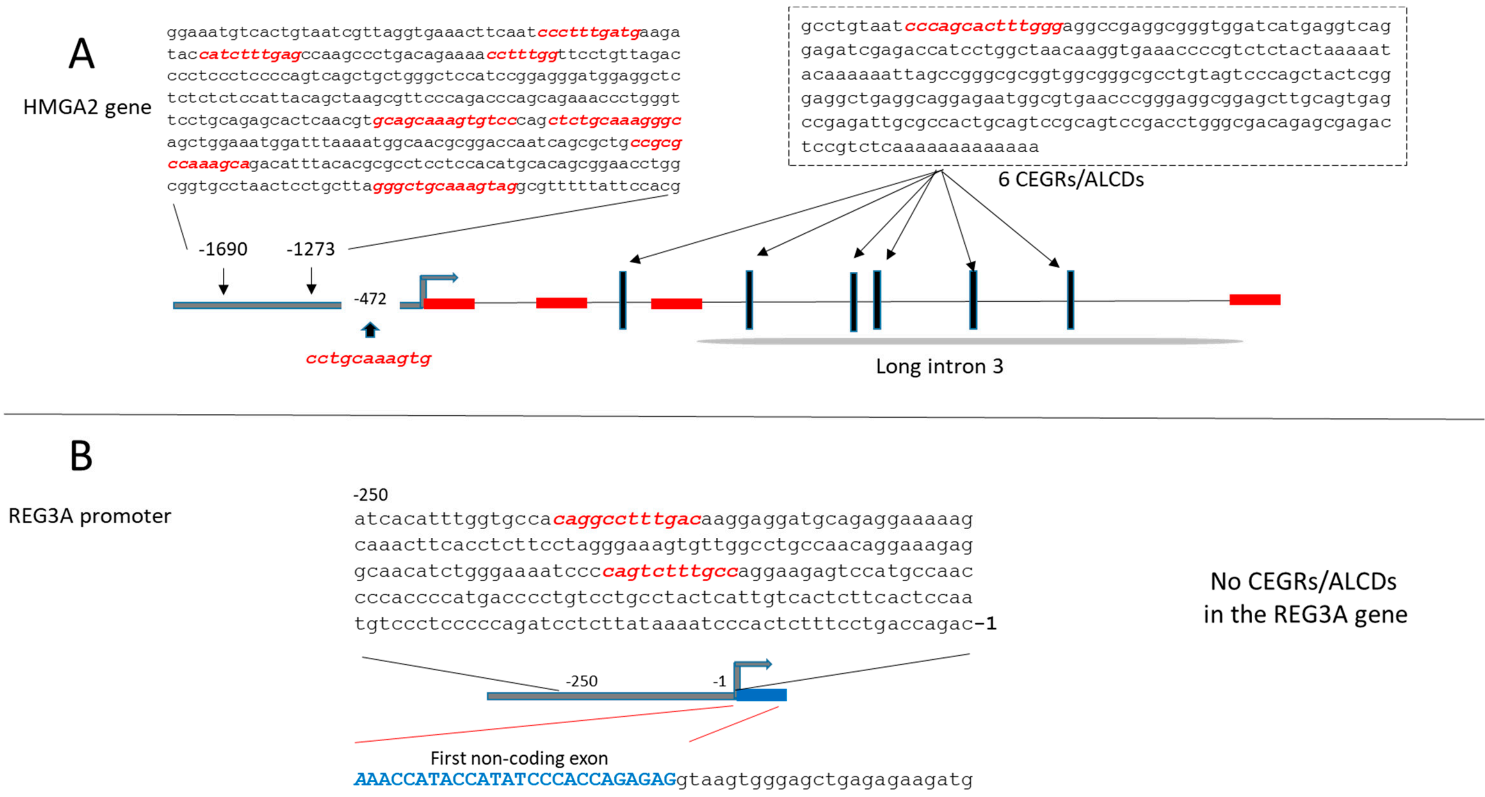

Figure S8. CEGRs/ALCDs and TCF4 binding sites in HMGA2 and REG3A genes. **A)** HMGA2 gene contains 8 TCF4 binding sites in the promoter and 6 CEGRs/ALCDs in introns 2 and 3. TCF4 binding sites are shown in red. **B)** REG3A promoter contains 2 TCF4 binding sites in close proximity to the start of transcription.

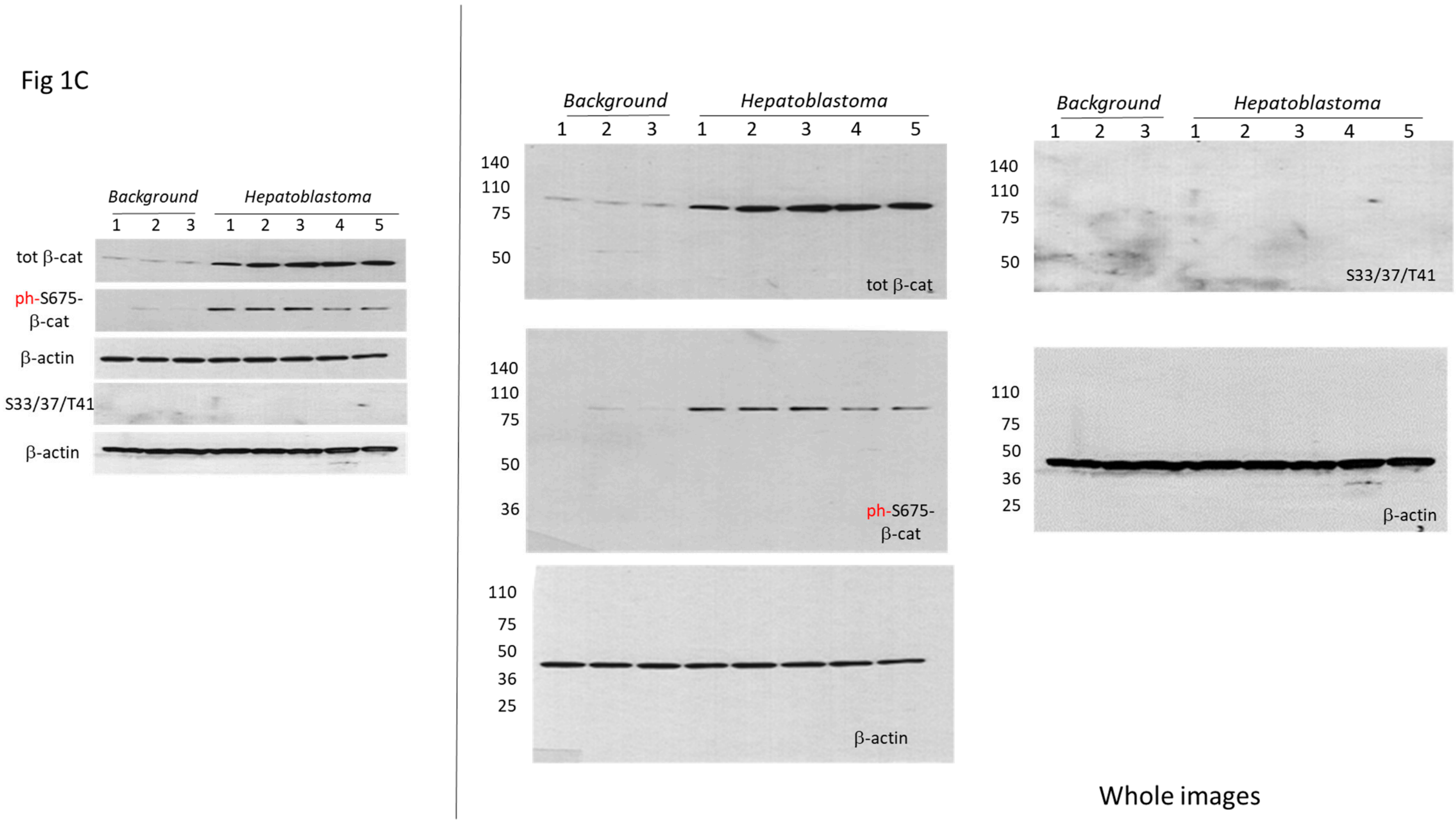

Fig 2A

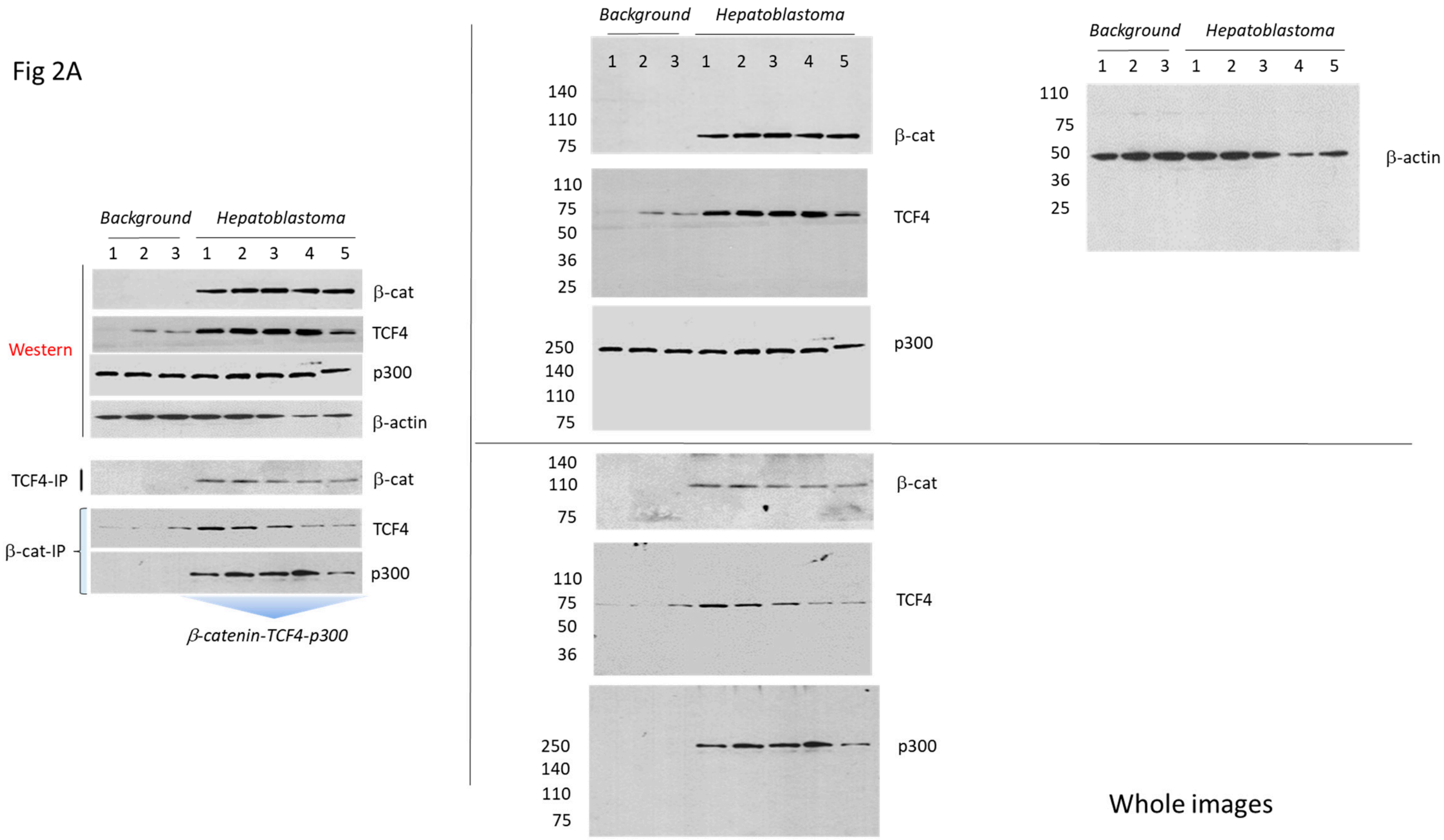
Background
Hepatoblastoma
1 2 3 1 2 3 4 5
Western
β-cat
TCF4
p300
β-actin
TCF4-IP
β-cat-IP
β-catenin-TCF4-p300
140
110
75
50
36
25
250
Whole images

Fig 2B

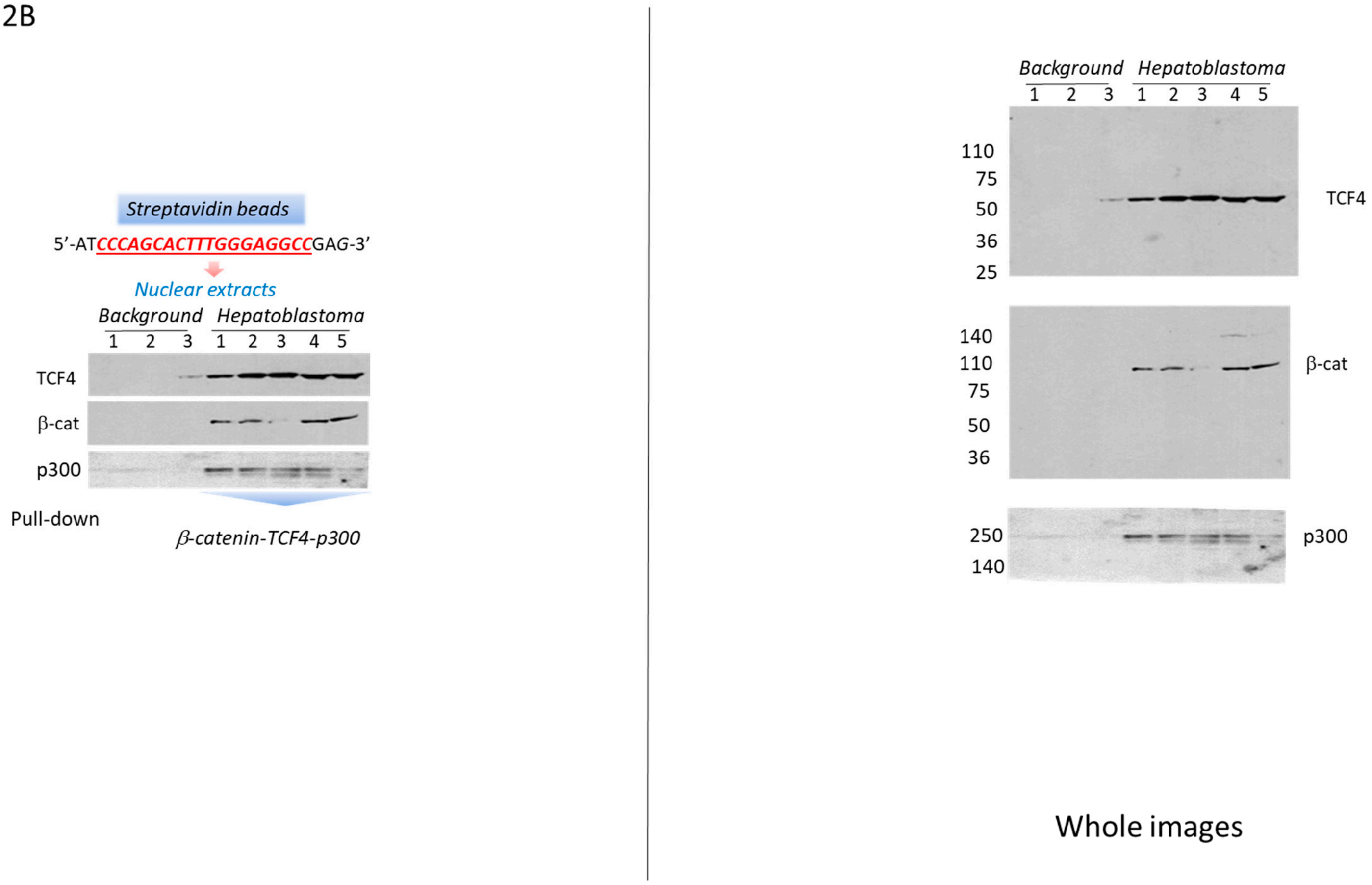
Streptavidin beads
5′-ATCCCAGCACTTTGGGAGGCCGAG-3′
Nuclear extracts
Background
Hepatoblastoma
1 2 3 1 2 3 4 5
TCF4
β-cat
p300
Pull-down
β-catenin-TCF4-p300
110
75
50
36
25
140
250
Whole images

Fig 3F

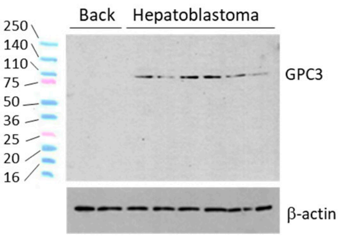

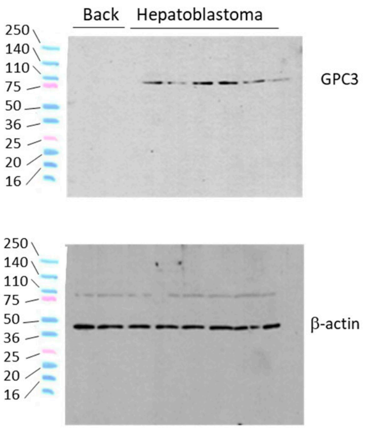

Whole images

Fig 3G

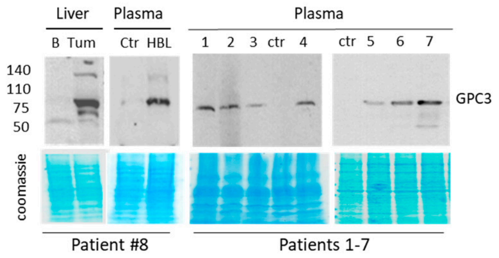

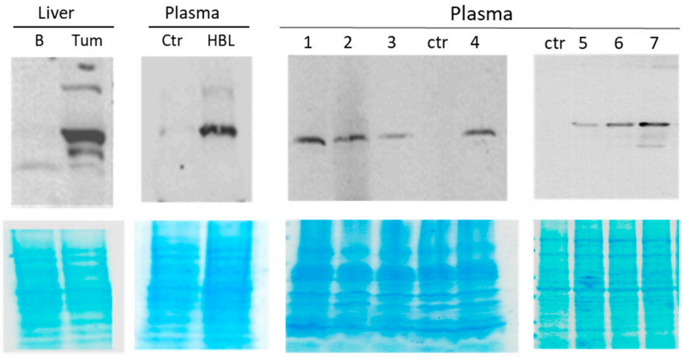

Fig 4A

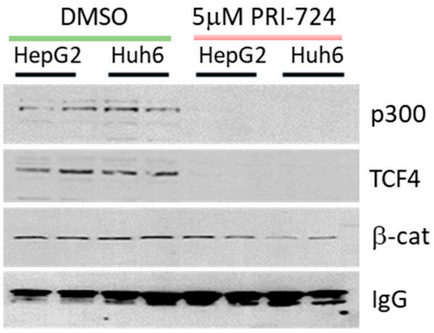

ph-S675-β-catenin IP

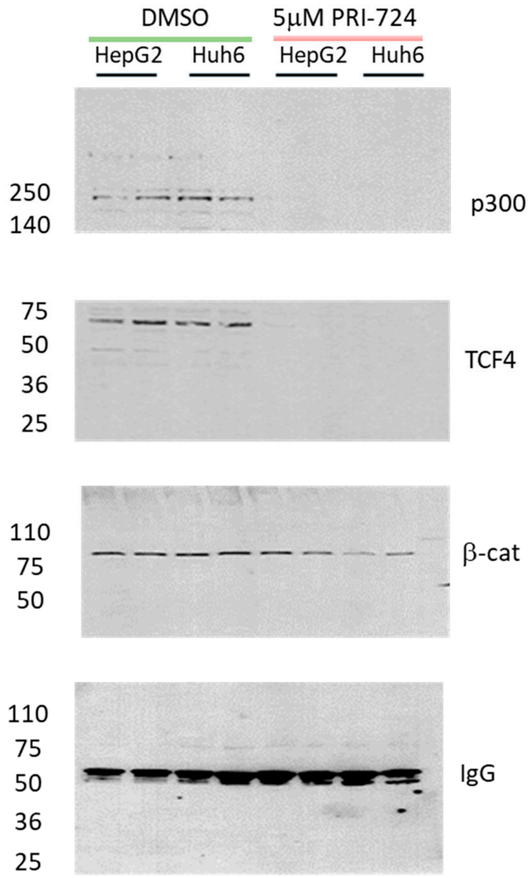

Whole images

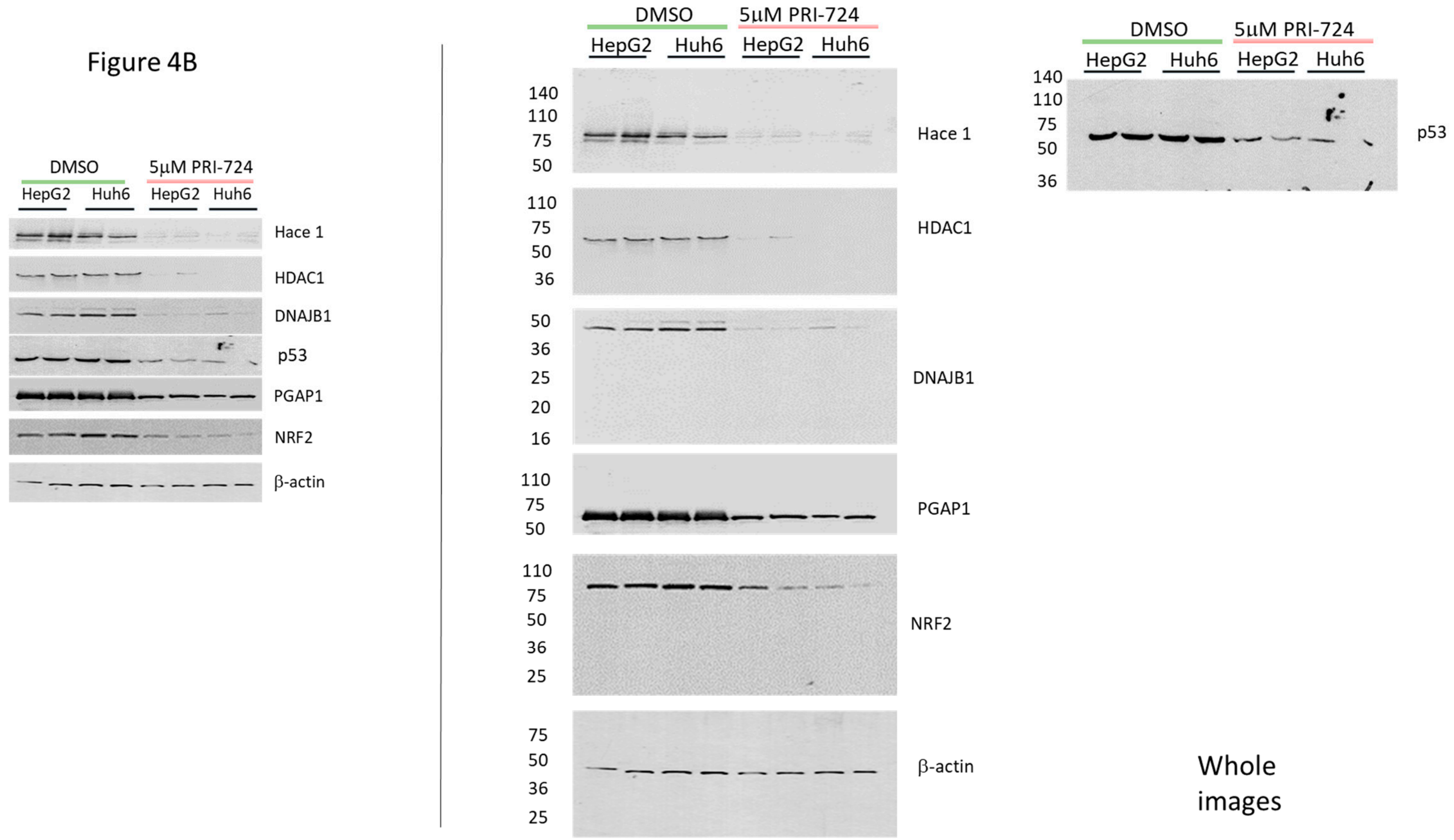
Figure 4B
DMSO
5µM PRI-724
HepG2
Huh6
Hace 1
HDAC1
DNAJB1
p53
PGAP1
NRF2
β-actin
140
110
75
50
36
25
20
16
Whole images

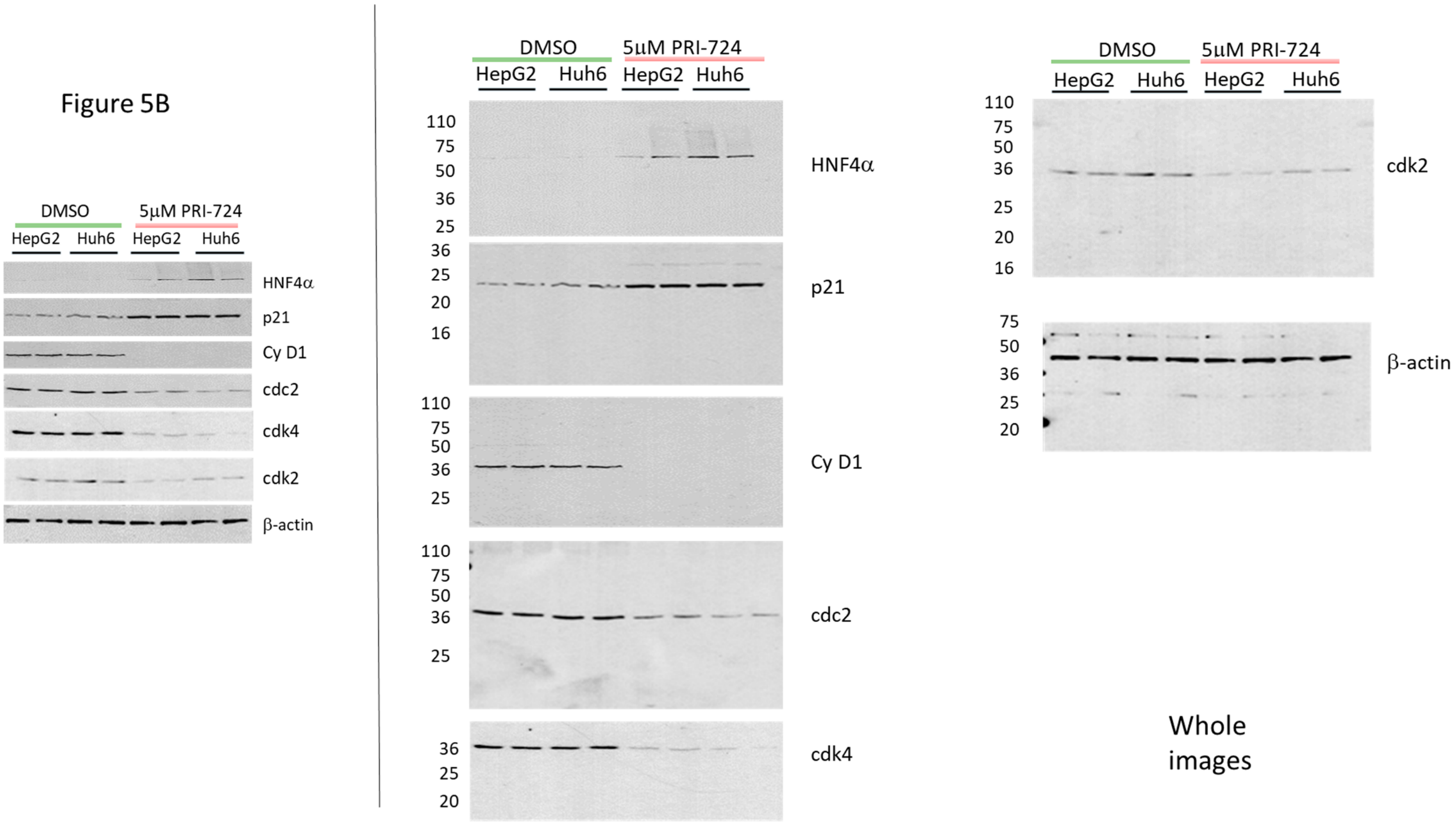
Figure 5B
DMSO
5µM PRI-724
HepG2
Huh6
HNF4α
p21
Cy D1
cdc2
cdk4
cdk2
β-actin
110
75
50
36
25
20
16
Whole images

Fig 6B

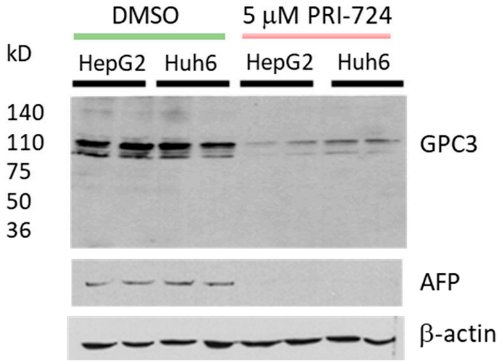

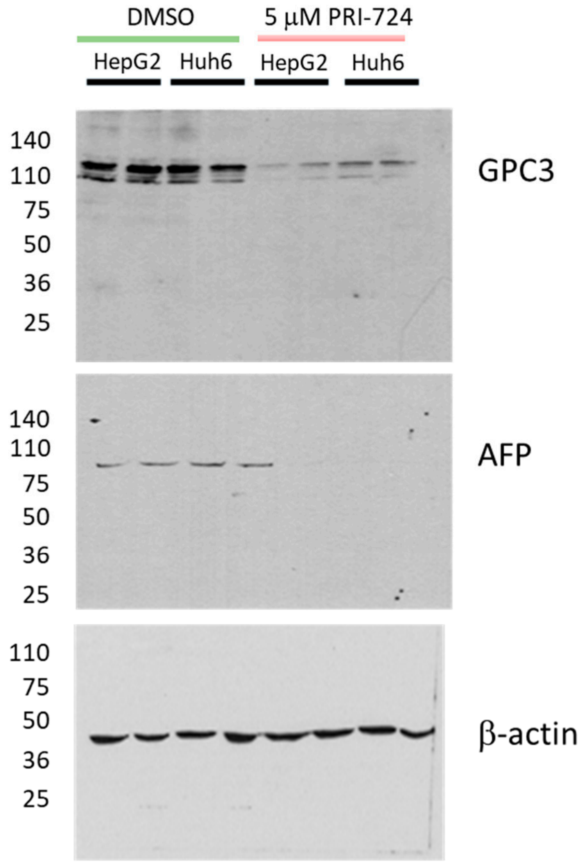

Whole images

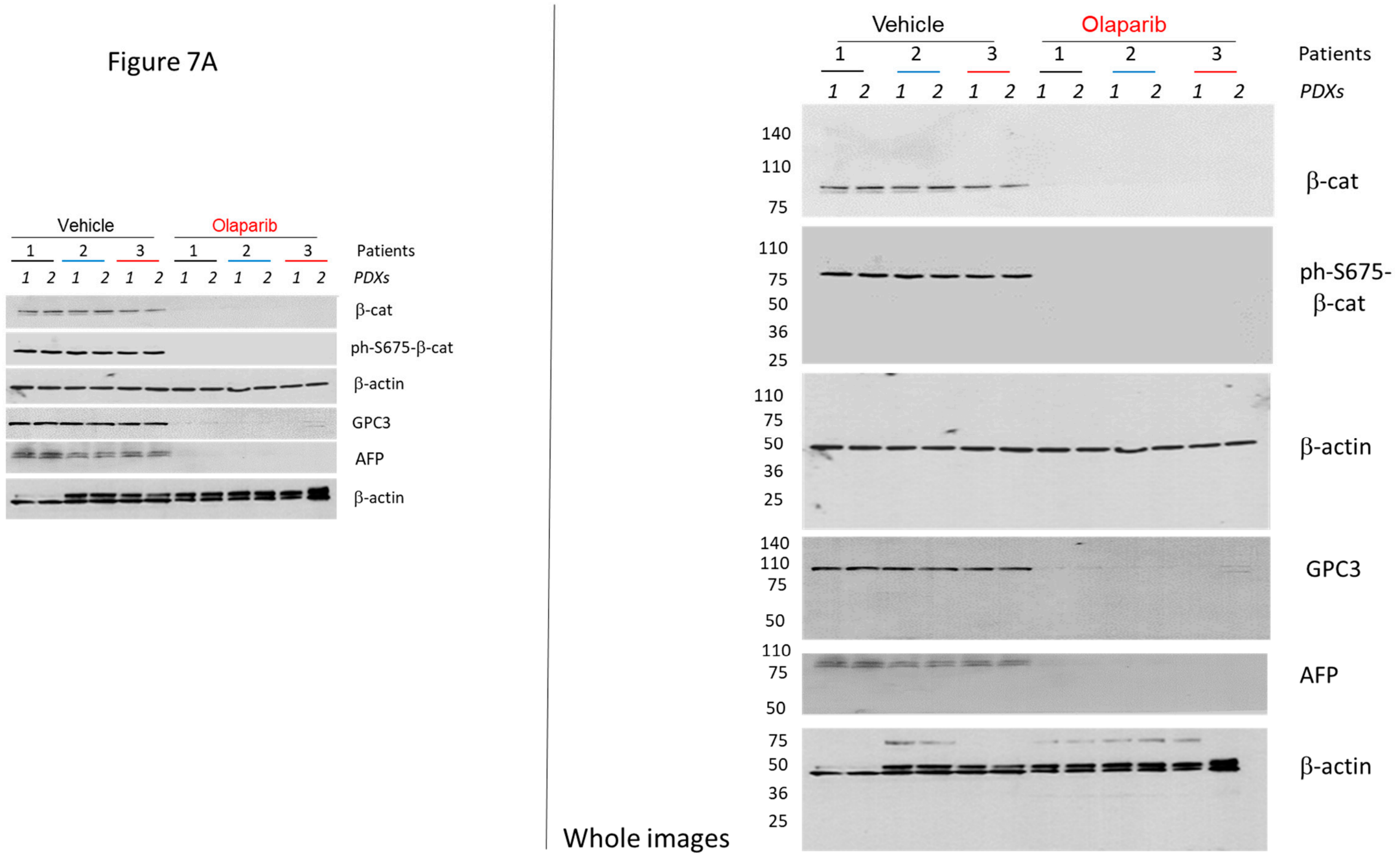

Figure S9. Uncropped Western blots.
